# TLR3 Agonist Poly-IC Induces IL-33 and Promotes Myelin Repair

**DOI:** 10.1371/journal.pone.0152163

**Published:** 2016-03-29

**Authors:** Chandramohan Natarajan, Song-Yi Yao, Subramaniam Sriram

**Affiliations:** Department of Neurology, Vanderbilt University, Nashville, Tennessee, United States of America; University of Utah, UNITED STATES

## Abstract

**Background:**

Impaired remyelination of demyelinated axons is a major cause of neurological disability. In inflammatory demyelinating disease of the central nervous system (CNS), although remyelination does happen, it is often incomplete, resulting in poor clinical recovery. Poly-IC a known TLR3 agonist and IL-33, a cytokine which is induced by poly-IC are known to influence recovery and promote repair in experimental models of CNS demyelination.

**Methodology and Principal Findings:**

We examined the effect of addition of poly-IC and IL-33 on the differentiation and maturation of oligodendrocyte precursor cells (OPC) cultured *in vitro*. Both Poly-IC and IL-33 induced transcription of myelin genes and the differentiation of OPC to mature myelin forming cells. Poly-IC induced IL-33 in OPC and addition of IL-33 to *in vitro* cultures, amplified further, IL-33 expression suggesting an autocrine regulation of IL-33. Poly-IC and IL-33 also induced phosphorylation of p38MAPK, a signaling molecule involved in myelination. Following the induction of gliotoxic injury with lysolecithin to the corpus callosum (CC), treatment of animals with poly-IC resulted in greater recruitment of OPC and increased staining for myelin in areas of demyelination. Also, poly-IC treated animals showed greater expression of IL-33 and higher expression of M2 phenotype macrophages in the CC.

**Conclusion/Significance:**

Our studies suggest that poly-IC and IL-33 play a role in myelin repair by enhancing expression of myelin genes and are therefore attractive therapeutic agents for use as remyelinating agents in human demyelinating disease.

## Introduction

Recognition of invading pathogens is mediated by the activation of innate and adaptive arms of the immune response.[1]. The cellular elements of the innate immune system consists of the pathogen associated molecular pattern (PAMP) receptors which recognize common structures present in bacteria and viruses. The PAMP receptors consists of 13 member of the Toll like receptor family and nucleotide binding and oligomerization domain (NOD)-like receptors (NLRs) and the retinoic acid-inducible gene I (RIG-I)-like receptors (RLRs) and nucleotide binding and oligomerization domain (NOD)-like receptors (NLRs) and the retinoic acid-inducible gene I (RIG-I)-like receptors (RLRs) [2–4]. The receptors which mediate innate immune response are expressed either on the cell surface or present in the cytosol of many cell types, including the cells of the CNS [1]. All PAMP receptors share a common purpose, which is to recognize the presence of danger, initiate mechanisms to eliminate the threat and ensure the survival of the organism.

TLR3 is the PAMP receptor for double stranded RNA (dsRNA). TLR3 is present in endosomes of antigen-presenting cells, endothelial cells and airway epithelium and glial cells of the CNS [5–9]. Poly-IC is a synthetic mimic of double stranded RNA and an agonist of TLR3 [10]. Activation of TLR3 by its ligand poly-IC, was protective in the experimental autoimmune model of demyelination, suggesting that the activation of inflammatory pathways by TLR3 paradoxically, reduced the inflammatory response in experimental models of CNS autoimmune disease [11]. One of the cytokines induced by poly-IC and thought to play an immunoregulatory function was IL-33. Poly-IC in comparison to other TLR ligands is a strong inducer of IL-33 in human peripheral blood lymphocytes. Also, administration of IL-33 reduced the severity of experimental allergic encephalitis (EAE) a mouse model of demyelinating disease which shares similarities with human multiple sclerosis [12, 13]. The therapeutic benefits of IL-33 were believed to be due to its ability to polarize macrophages to the M2 phenotype. M2 subtype of macrophagesare known to secrete anti-inflammatory cytokines and promote repair [14–16].

In addition to its immunoregulatory properties, other experimental studies had suggested that IL-33 may be a growth factor involved in the recruitment and maturation of oligodendrocytes [17]. Histological lesions in areas of spinal cord following trauma had shown high levels of IL-33 expression in regions showing increased expression of oligodendrocyte precursor cells (OPC). In models of traumatic spinal cord injury, recruitment of OPC and repair was decreased in IL-33-/- mice suggesting a neuro-reparative role for IL-33. These observations, led us to speculate that the inflammatory signals induced by TLR3 agonists and the induction of IL-33 can directly influence oligodendrocyte recruitment and maturation and thereby promote remyelination. We therefore examined the effect of both poly-IC and IL-33 on maturation and differentiation of oligodendrocyte precursor cells *in vitro* and the effect of poly-IC on demyelination/remyelination [18, 19].

## Materials and Methods

### Rats

Sprague-Dawley rats (Charles River Laboratories) were used for all studies. One to two day old new born pups from pregnant mothers were used for the isolation of oligodendrocytes.

### Reagents

Anti-O4, anti-MBP, anti-Olig2, and anti-S-100 antibodies were purchased from EMD Millipore (Billerica, Massachusetts, USA). CD68 (ED1) was purchased from ADb Serotech (Raleigh, NC). Anti MBP antibody was purchased from Millipore (Billerica, MA) and anti-CC1 antibody was purchased from Abcam (Cambridge, MA). SYBR Green master mix solution was purchased from Thermo scientific (Swedesboro, NJ). Triiodothyronine (T3), lysolecithin (LPC) and chloroquine were purchased from Sigma-Aldrich (St. Louis, MO). SB203580 and poly-IC was purchased from Invitrogen (San Diego, CA). Recombinant rat IL-33 was purchased from ProSpec-TanytechnoGen (East Brunswick, NJ). Bovine-FGF and PDGF were purchased from Pepro Tech (Rocky Hill, NJ). Anti-IL33 antibody was purchased from Enzo Life Sciences, Inc (Farmingdale, New York). Alexa-488 or 555 conjugated anti-rabbit or anti-mouse second antibodies were purchased from Life technologies (Grand Island, NY). Percoll solution was purchased from GE Healthcare Bio-Sciences (Pittsburgh, PA).

### Preparation of oligodendrocyte precursor cells

Rat oligodendrocyte progenitor cells (OPC) were purified from cerebral hemispheres, following as per our previously published protocols [4, 20]. Following this method of purification, we have previously shown that > 95% of cells belong to one of three groups, A2B5^+^, Olig2^+^MBP^-^ and O4^+^MBP^+^ [4].

### Western blot analysis of myelin basic protein synthesis

To evaluate the role of poly-IC induced synthesis of myelin proteins, we performed western blot analysis for myelin basic protein (MBP) in OPC. Postnatal day 3 or 4 old OPC (unless otherwise mentioned, all *in vitro* experiments performed in OPC were started in postnatal day 3 or 4 were incubated with increasing doses of poly-IC or IL-33 for a period of 10d in OPC differentiation culture medium (PDGF and bFGF free). Every third day, fresh medium containing either poly-IC or IL-33 was added to replace old medium. After 10 days of treatment, cells were washed with PBS, harvested and lysed with radio-immunoprecipitation assay (RIPA) buffer containing mammalian protease inhibitor cocktail (Sigma, St. Louis, MO). Equal amount of cell lysates was resolved in 10% SDS-PAGE. Proteins were then transferred onto PVDF membrane. Blot was blocked with 5% non-fat dry milk for 30 min at room temperature and incubated with anti-MBP rabbit polyclonal antibody (1:1000) in 3% milk overnight at 4°C. The next day, blot was washed and incubated with HRP conjugated secondary antibody for 2 h at room temperature and the blot was then developed using enhanced chemi-luminescent (ECL) reagents.

To examine poly-IC induced IL-33 synthesis *in vitro*, OPC were treated with increasing doses of poly-IC for 10 days and the cells were harvested. The cytosolic and nuclear fraction were prepared using the cytosolic (10 mM HEPES-pH7.9, 10mM KCl, 0.1mM EDTA, 0.1 mM EGTA, 0.4% NP-40) and nuclear (20mM HEPES pH 7.9, 400mM NaCl, 1mM EDTA, 1mM EGTA) extraction buffers that contained 1 mM dithiothreitol, 0.5 mM PMSF and protease inhibitor cocktail.

Proteins in the nuclear fractions were analyzed for poly-IC induced IL-33 accumulation. In addition, to examine whether IL-33 is playing role in MBP synthesis, we also treated OPC with increasing doses of rat recombinant IL-33 for 10 days and the cell lysates were tested for MBP synthesis by western blot analysis.

To quantitate the phosphorylation of p38MAPK, OPC were stimulated with doses of either poly-IC or IL-33 for 8 min and immediately washed and frozen in liquid nitrogen for a few min. The cells in the culture dishes were lysed with RIPA buffer containing protease inhibitor and mammalian phosphatase inhibitor cocktail 3 (Sigma, St. Louis, MO). Samples were resolved in SDS-PAGE and the blot was incubated with 5% BSA for 30 min at room temperature. Blot was then incubated with anti-phopho p38MAPK rabbit polyclonal antibody (SC-17852-R; 1:1000) (Santa Cruz, La Jolla, CA). β-actin and total p38 (SC-535; 1:1000) were treated as internal loading controls. Images of western blot bands were scanned and quantified using Bio-Rad imaging software.

### Immunocytochemistry

Postnatal day 3 or 4 rat OPC (2 x 10^5^) were plated into poly-D-lysine (100 μg/ml of sterile water; the wells were twice coated for 1 h and were air dried for 1.5 h under UV light in the laminar flow hood, at room temperature) coated 8 well chamber slides (LabTech, Naperville, IL) in culture medium containing platelet derived growth factor (PDGF) and bovine fibroblast growth factor (b-FGF). The next day, old medium was replaced with fresh differentiation medium (without PDGF and βFGF). Poly-IC or rat recombinant IL-33 was added until the end of the experiments and the plates were incubated at 37°C with 5% CO_2_. After the experimental periods, chamber slides were fixed with 4% paraformaldehyde (Electron Microscopy Sciences, Washington, PA) in PBS for 10 min at room temperature, washed 3 x 5 min with PBS and permeabilized with 0.2% Triton-X 100 for 5 min at room temperature. Wells were again washed 3 x 5 min with PBS and blocked with 1% BSA containing 5% normal goat serum for 30 min at room temperature. Staining was performed using anti-O4 mouse monoclonal, and anti-myelin basic protein rabbit polyclonal antibodies and the chamber slides were incubated overnight at 4°C. The next day, wells were washed 3 x 5 min with TBS (10mM Tris-HCl; pH 8.0, 150mM NaCl) and blocked with 1% BSA in PBS for 20 min at room temperature before the addition of secondary antibodies. Blocking solution was replaced with 1% BSA in PBS containing 1:1000 dilution of Alexa 488 conjugated goat anti-mouse IgM (for O4) and Alexa 555 conjugated goat anti-rabbit IgG (for myelin basic protein) antibodies, incubated for 2 h at room temperature. Chambers were again washed 3 x 5 min with TBS and mounted using Vectashield mounting medium containing DAPI.

### *In Vivo* injection of lysolecithin into corpus callosum and poly-IC treatment

Sprague-Dawley rats were used in these experiments, with all animal protocols approved by Vanderbilt University institutional Animal Care and Use Committee. Two month old rats were anesthetized and positioned in a small-animal stereotaxic apparatus (David Kopf Instruments, Tujunga, CA) to conform to the brain atlas [21]. Microinjection of lysolecithin into the corpus callosum was performed with a 32-gauge needle through a dentist’s burr hole. To perform the injection, the following coordinates were used: 1 mm posterior from bregma, 1 mm lateral from the sagittal suture, and 3.3–3.5 mm below the dura mater. The rats were injected with 5μl of 1% lysolecithin in sterile saline solution or saline solution alone, using microinjection pumps over 15 min and the needle was held for an additional 10 min after injection. Rats were injected with either equal volume of saline or poly-IC (sc injection of 10 μg/Kg/everyday in 400ul saline solution) and the treatments were started at the beginning of lysolecithin microinjection and continued until day 21. We sacrificed the experimental rats on day 3, 7, 14 and 21 after lysolecithin injection and the brains were used for pathological studies.

### Immunohistochemistry

To perform immunostaining in paraffin embedded brain tissues, rats were perfused with 4% formaldehyde/4% sucrose in PBS. Brains were removed and post-fixed in 10% formaldehyde for 24 h. The injection site was identified by placing the brain in a brain mold and a 2 mm thickness of brain which spanned 1 mm anterior and 1 mm posterior to the injection track was cut and embedded in paraffin. Serial coronal sections (8–10μm thickness) of the brain were cut and subjected to histochemical (Luxol-Fast blue—Periodic-Acid Schiff) staining. For immunostaining, sections were de-paraffinized and antigen retrieval was performed using antigen retrieval solution (Vector Laboratories) for 20 min in a steamer. Sections were washed 2 x 5 min with water and thereafter with TBS-T (TBS containing 0.05% of Tween-20). Sections were blocked with blocking solution (5% FBS/1% goat serum in TBS containing 0.2%Triton X-100 for 60 min at room temperature. The sections were then incubated with respective primary antibodies diluted in TBS-Triton X-100 buffer (Olig2, 1:100; S100, 1:200; CD68, 1:200, iNOS 1:100; CD 206 1:50; Arginase 1:250 and IL-33 1:500) and incubated overnight at 4°C. The next day, sections were washed 5 x 5 min with TBS-T buffer and incubated with Alexa-488 or 594 conjugated secondary antibodies (1:1000) for 60 min at room temperature. The stained sections were imaged using a fluorescence microscope (Olympus Ax70A) with a CCD camera (Q color 3, Olympus, USA) and Image J software was used to analyze the fluorescent staining.

### Image Analysis

To quantify the positive cells following staining for IL-33, Olig2, S-100, CC-1, MBP and CD68, three regions were chosen for these analyses; the cingulum region of injection site, the cingulum region of the contralateral hemisphere and the center region of corpus callosum (Zhang, et. al, 2013). In each group, three areas of each specimen from different animals were analyzed and the images with 2,048 x 1,536 pixel resolutions were acquired at 10x or 20x magnification with a CCD camera. Using Image J software, color images were first converted to grayscale images and enhanced using a median filter. The interval of grey shades, corresponding to the staining, was defined and the area occupied by the staining was extracted. The threshold operation converted foreground pixels into black color and background pixels into white color. Thus, the binary image represented the analyzed image. The area of positive immunofluorescence was estimated by the quantity of black pixels and presented as percentage of black pixels in the binary image. The results was represented as the ratio of total imaging density of specific cell staining (IL-33, Olig2, S-100 and CD68 positive cell) divided by total imaging density of total cells (DAPI staining). The percentage of IL-33 specific cell staining results was represented as the ratio of total number of double positive cell divided by total single positive cell number.

In Luxol Fast Blue-Periodic Acid Schiff (LFB-PAS) stained slides, one threshold recognizes red (PAS) and another recognizing blue (LFB). The thresholding operation converted blue-positive into black color, and red (PAS stained) pixels into white color. The area of positive reaction was estimated by the number of black pixels and presented as the percentage of black pixels in the binary image. Three regions chosen from corpus callosum and the ratio of positive and negative staining from each region is calculated.

The cells expressing iNOS, CD206 or Arginase1 were performed using 10X objective images. The pictures were taken from at least six consecutive brain sections which included the injected and non-injected side. The number of immunopositive cells (iNOS, CD206 or Arginase 1) per mm2 was obtained with ImageJ- Plugin software.

### Quantitative real time PCR

Quantitative RT-PCR (qRT-PCR) analysis was performed in OPC stimulated with either poly-IC or IL-33 to examine the expression of myelin related genes as well as few transcription elements that bind to the MBP promoter region. Postnatal day 3 or 4 d old OPC (3 x 10^6^) were treated with either poly-IC or IL-33 in 35 mm tissue culture dishes for 7 d. Fresh medium containing either poly-IC or IL-33 was replaced with every third day. On the 7^th^ day after treatment, cells were washed with PBS and lysed with TRIzol reagent (Life technologist, Grand Island, NY) and total RNA was isolated following manufacturer’s instructions. In some cases, total RNA was isolated 4h after the treatment of either poly-IC or IL-33 and compared with the 7^th^ day treatments. qRT-PCR was performed using SYBR-Green master mix solution in the Bio-Rad CFX Real Time PCR instrument with specific primer sequences (Table 1 RT-PCR primers sequences). Data were analyzed using Bio-Rad CFX manager version 3.1 software. The comparative CT method was used to quantitative real-time PCR data and expression as fold change by the equation (Fold change = 2^-ΔΔCT^).

**Table 1:** Primer Sequences for Real-Time PCR.

Name	Sequence	GenBank Accession No	Products Size
GAPDH	FW 5’-CCATCAACGACCCCTTCATT-3’ FW 5’-GACCAGCTTCCCATTCTCAG-3’	NM_017008	110
MBP	FW 5’- AAGAACTACCCACTACGG-3’ FW 5’-CCTCTCCCCTTTCCTTG-3’	NM_001023291	133
PLP	FW 5’-TCAGCCGCAAAACAGACTAG-3 FW 5’-CACTCCAAAGAAACACAATCCAG-3’	NM_030990	143
MOG	FW 5’-TAAAGATGGCCGGTGTGTGG-3’ FW 5’-TCATCCCCAACTAAAGCCCG-3’	NM_022668	147
Pura	FW 5’-AGCGTTTCTACCTGGACGTG-3’ FW 5’-CGTAGTGCTCGATGAAGTCG -3’	XM_003751762	153
Sox10	FW 5’-GCTATCCAGGCTCACTACAAG-3’ FW 5’-ACTGCAGCTCTGTCTTTGG-3’	NM_019193	148
SP1	FW 5’-TGCCCCTATTGTAAAGACAGTG-3’ FW 5’-ACGGAGATGTGAGGTTTTGC-3’	NM_012655	117
IL-33	FW 5’-GAACCCGCCAAAAGATATTCAC-3’ FW 5’-AAGTTCCTTGGATACTCAGTGTG-3’	NM_001014166	148
iNOS	FW 5’-AGCATCCCAAGTACGAGT-3’ FW 5’-AATCTCCCTGCCCATGTA-3’	NM_012611	140
CD206	FW 5’-CCCTGCTCCTGCTTTTATCT-3’ FW 5’-CTGAACGGAGATGGCGCTTA-3’	NM_0011061232	125
Arginase 1	FW 5’-ATATCTGCCAAGGACATCGTG-3’ FW 5’-AGGTCTCTTCCATCACTTTGC-3’	NM_017134	142

### Statistical analyses

All data are presented as mean ± SD. Multiple comparisons were made using one-way analysis of variance (ANOVA) followed by the Newman—Keuls multiple comparison test. Comparisons between treatment groups versus controls were analyzed by Student's paired *t*-test. Statistical analyses were performed using Prism 5 (GraphPad Software) and p-value less than 0.05 was considered statistically significant.

### Animal Care protocol

All experimental procedures involving animals were carried out in accordance with the recommendations in the NIH Guide for the Care and Use of Laboratory Animals. The Institutional Animal Care and Use Committee of Vanderbilt University Medical Center, (protocol number# M/13/130) approved the study. All animals were housed in temperature and humidity controlled rooms maintained on a 12hr light dark cycle. Animals were euthanized using CO2 narcosis followed by decapitation. All efforts were made to minimize the number of animals used and to ameliorate their suffering.

## Results

### Culture of OPC in the presence of poly-IC induces expression of myelin basic protein and transcription of myelin genes

**Fig 1:**
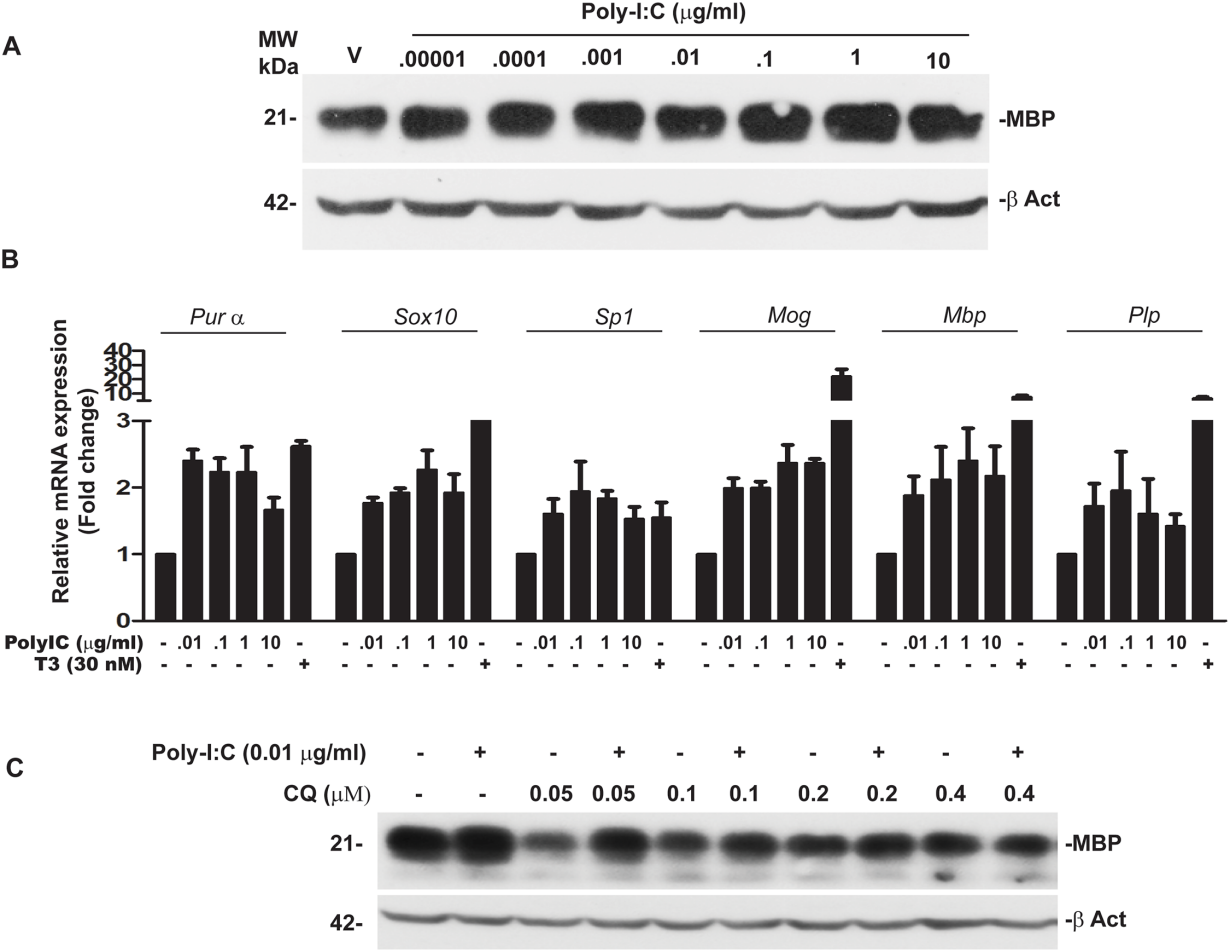
Poly-IC induces MBP expression and myelin related genes in OPC, (representative of four separate experiments). (A) Western blot analysis performed in OPC show a dose dependent increase in MBP level following culture with poly-IC. (B) QRT-PCR assay shows poly-IC induced upregulation of some major genes associated with myelin synthesis as well as the *Mbp* promoter region binding transcription factors *Pur α*, *Sox10* and *Sp1*. Triiodothyroxine was used as a positive control for the study. (C) Western blot shows that chloroquine (CQ) pretreatment effectively blocks poly-IC induced MBP synthesis *in vitro*.

Western blot analysis of cell lysates from OPC treated with poly-IC showed a dose dependent increase in myelin basic protein (MBP). Maximal induction of MBP (1.62 ± 0.36 fold over vehicle was seen when 0.1 μg/ml of poly-IC was added to the culture (Fig 1A). We also quantitated mRNA of *Mbp*, myelin oligodendrocyte protein (*Mog*) and proteolipoprotein (*Plp*) along with mRNAs of *Purα*, *Sox10* and *Sp1* in OPC cultured with poly-IC. *Purα*, *Sox10* and *Sp-1* are transcription elements which bind to the promoter region of Mbp to initiate gene expression [22]. As shown in Fig 1B, addition of poly-IC resulted in a 2.39 ± 0.69 fold increase in *Mbp*, *2*.*36* ± 0.38 fold in *MOG and* 1.95 ± 0.82 fold increase in *Plp* mRNA when compared with vehicle treated controls. We also observed a 2.2± 0.53 fold increase in *Purα*, 2.3 ± 0.4 fold increase in *Sox10* and 1.8 ± 0.16 fold increase in *Sp1* (Fig 1B).

TLR3 is the endosomal receptor for poly-IC and chloroquine is a known inhibitor of endosomal activity [23]. To show that TLR3 activation is required for the expression of MBP we examined the expression of MBP in OPC cultured with poly-IC in the presence of chloroquine. Addition of chloroquine, to OPC cultures in the presence of poly-IC resulted in 92% decrease in the expression of MBP (Fig 1C).

### Poly-IC induces maturation of OPC

**Fig 2:**
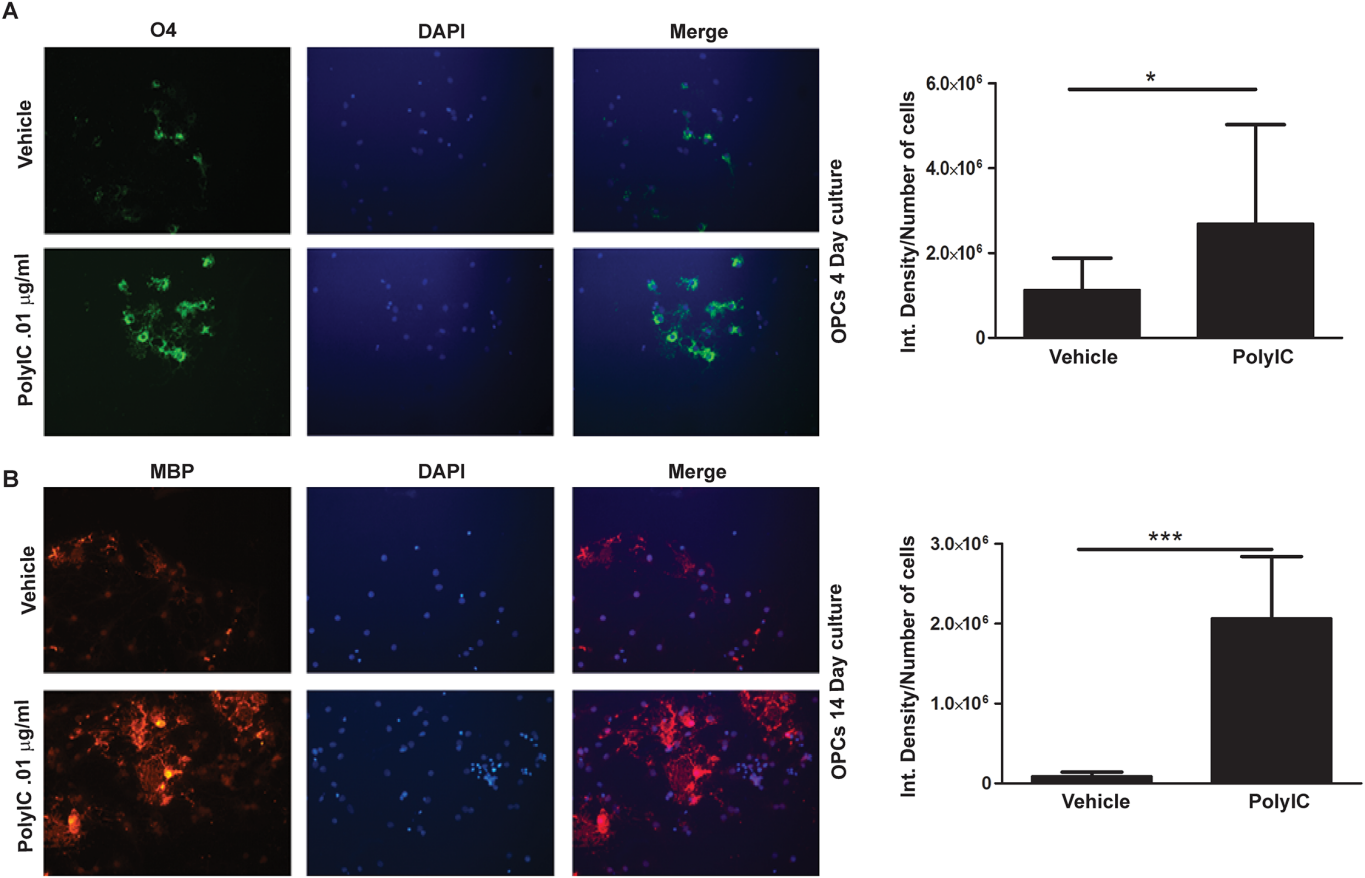
Poly-IC treatment induces earlier maturation of OPC *in vitro*. Immunostaining and quantitative image analysis of the expression level of maturation markers O4 (A) and MBP (B) in OPC. OPC in cultures were treated with 0.01 μg/ml of poly-IC for 4 d (A) and 14 d (B). Corresponding bar graphs represent quantitative analysis of O4 (A) and MBP expression (C & D) *, p<0.05.

We examined the effect of poly-IC on the differentiation of OPC. Expression of O4 antigen, is seen early in the maturation process of OPC and persists through the period of oligodendrocyte maturation, while the expression of MBP is seen later. Addition of poly-IC to OPC cultured for four days increased O4 expression when compared with vehicle treated controls (p<0.02, Fig 2A). In OPC which were cultured for 14 days, time necessary for MBP expression, a 23.2 fold increase of MBP was seen when 0.01μg/ml of poly-IC, (p, 0.01, Fig 2C). Mature OPC’s expressing MBP showed a web like extension of its processes, which were more prominent in poly-IC treated cells (Fig 2B).

### Poly-IC induces IL-33 which is a growth factor for oligodendrocyte differentiation

**Fig 3:**
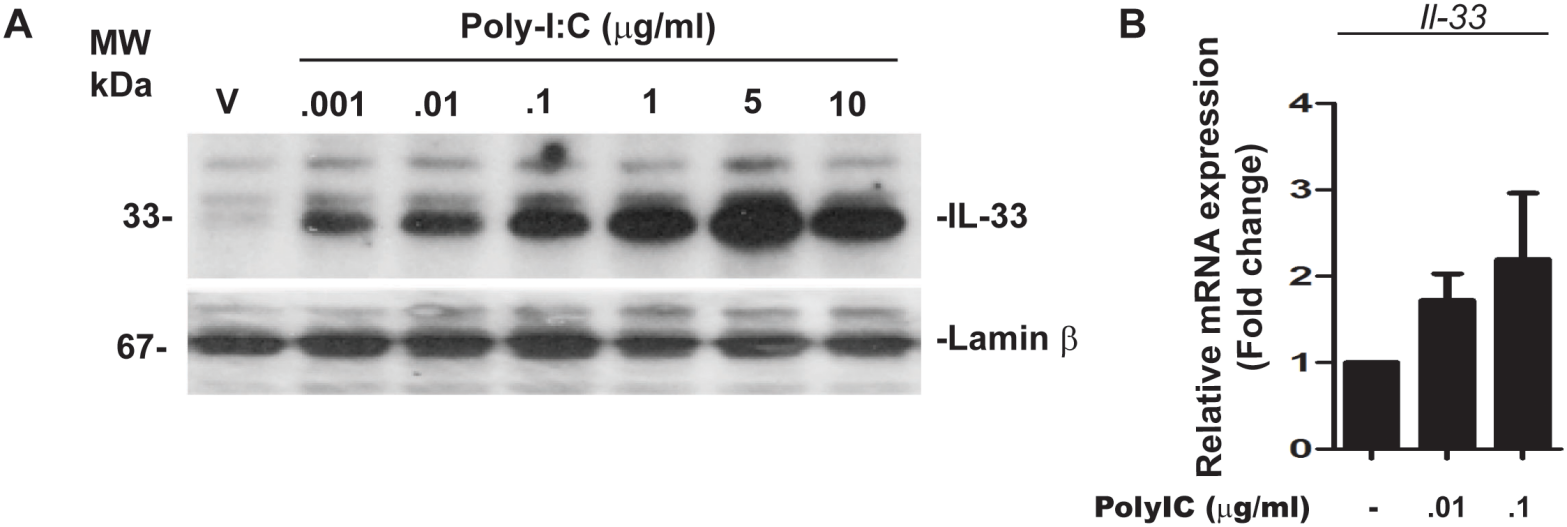
Poly-IC induces intranuclear accumulation of IL-33 in OPC (representative of three experiments). (A) Western blot analysis shows dose dependent accumulation of intranuclear IL-33 in poly-IC treated OPC *in vitro*; (B) Quantitative RT-PCR analysis represents poly-IC induced IL-*33* mRNA accumulation.

We have previously shown that, in human PBMC, addition of poly-IC to *in vitro* cultures results in the induction of IL-33 [13]. Since IL-33 is an intranuclear cytokine, we probed for the expression of IL-33 using nuclear extracts of OPC. Addition of poly-IC resulted in a dose dependent increase in IL-33 expression, which was maximal (3.24 ± 0.76 fold increase over vehicle) at 5 μg/ml concentration of added poly-IC (Fig 3A). In addition, qRT-PCR analysis revealed that OPC treated with 0.1 μg/ml of poly-IC for 7 days increased the level of IL-33 message to 2.19 ± 1.08 fold over vehicle treated control cells (Fig 3B).

**Fig 4:**
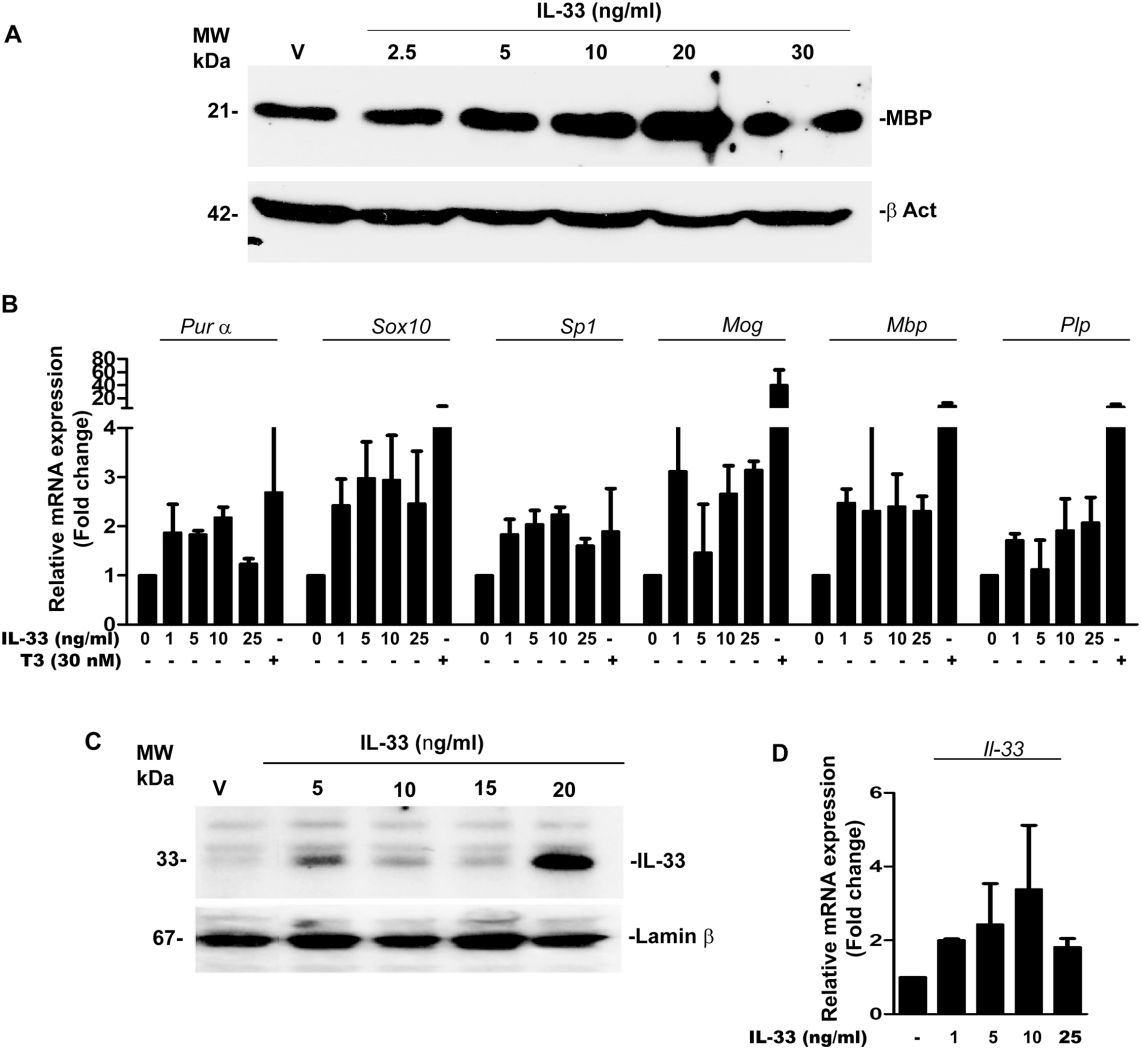
Induction of MBP in OPC treated with rIL-33 *in vitro* (representative of three experiments). (A) Western blot shows the dose dependent increase in MBP synthesis following culture with rIL-33; (B) QRT-PCR showing the expression of myelin genes and transcription factors *Purα*, *Sox10* and *Sp1*; (C) Western blots of nuclear extracts of OPC showing autocrine regulation of IL-33 by exogenously added IL-33; (D) Quantitative RT-PCR analysis represents IL-33 induced *IL-33* mRNA accumulation.

We next wanted to examine whether the addition of recombinant IL-33 (rIL-33) in OPC culture has any effect on inducing MBP synthesis and activating myelin related genes *in vitro*. OPC were cultured in the continued presence or absence of rIL-33 for 10 days. OPC treated with rIL-33 showed a 3.4 ± 1.6 fold induction of MBP synthesis *in vitro* (Fig 4A). Also rIL-33 induced mRNA expression of major myelin related genes in a manner analogous to that seen when poly-IC was added to culture. As shown in Fig 4B, addition of 10 ng/ml of rIL-33 induced expression which was 2.4 ± 0.9 fold in *Mbp*, 2.7± 0.8 fold of *Mog*, 1.9 ± 0.9 fold of *Plp*, 2.2 ± 0.3 fold of *Pur α*, 2.9 ± 1.2 fold of *Sox10* and 2.2 ± 0.2 fold of *Sp1* (Fig 4B).

IL-33 also acted in an autocrine manner to increase nuclear expression of IL-33. Addition of exogenous IL-33 to OPC culture resulted in a dose dependent increase in IL-33 in the nucleus. This was further confirmed using quantitative real time PCR which showed a 3.8+/- fold increase in mRNA levels of IL-33 (Fig 4C and 4D)

**Fig 5:**
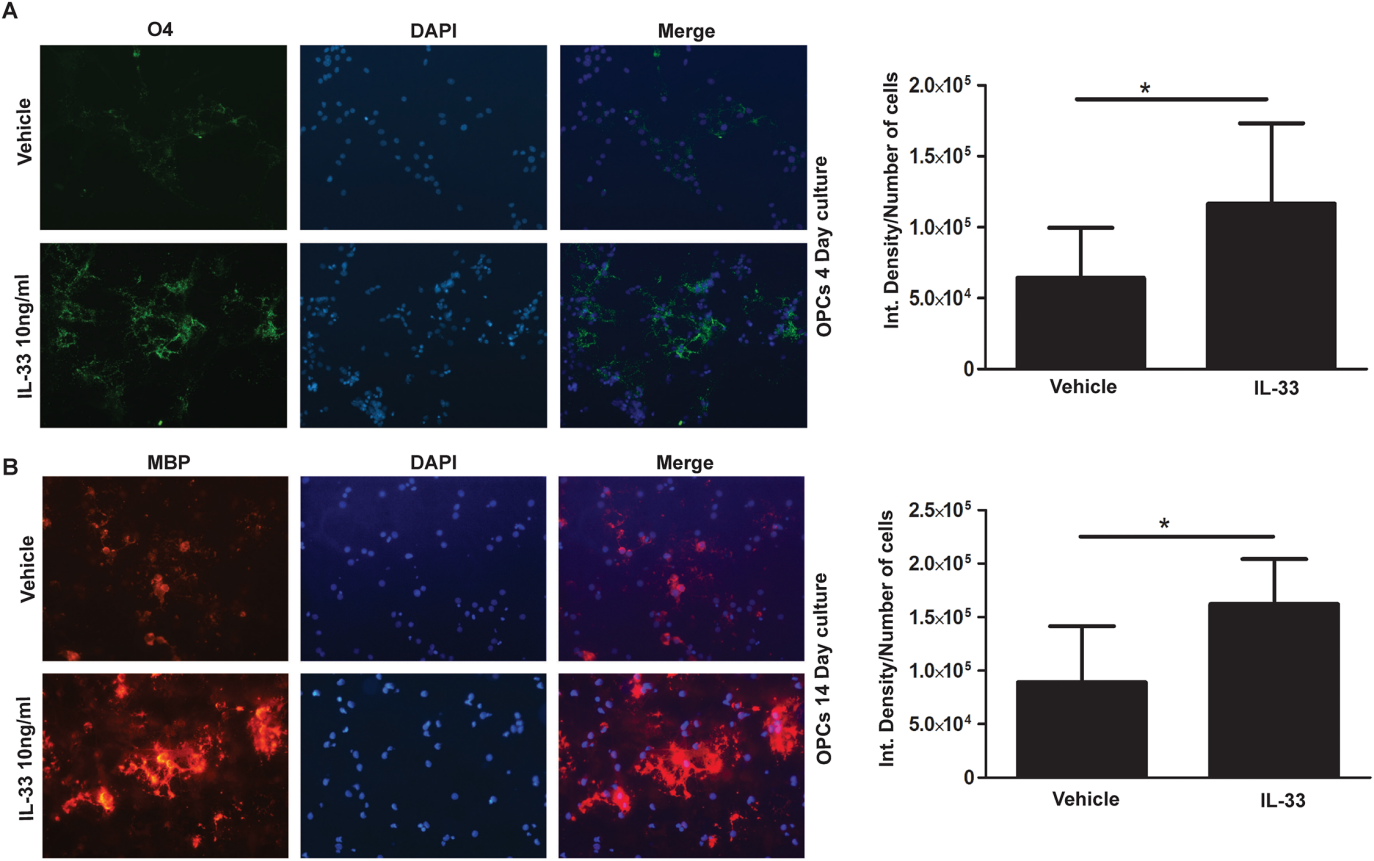
IL-33 treatment stimulates earlier differentiation and maturation of OPC *in vitro*. Immunocytochemical studies show the increase in the number of O4 expressing cells on day 4 post isolation of OPC, in the presence of rIL-33 (A) and MBP expressing cells in on day 14 (B). Bar graphs on the right show the respective quantitation of O4 and MBP.*, p<0.05.

Maturation of OPC was also promoted by IL-33. As shown in Fig 5, addition of 10 ng/ml rIL-33 to OPC cultures increased the level of O4 expression when compared to vehicle treated controls (Fig 5A, p< 0.05). OPC cultured for 14 days with poly-IC, increased the expression of MBP (p< 0.05) when compared to vehicle treated controls (Fig 5B).

### Poly-IC and IL-33 activate p38 MAPK

**Fig 6:**
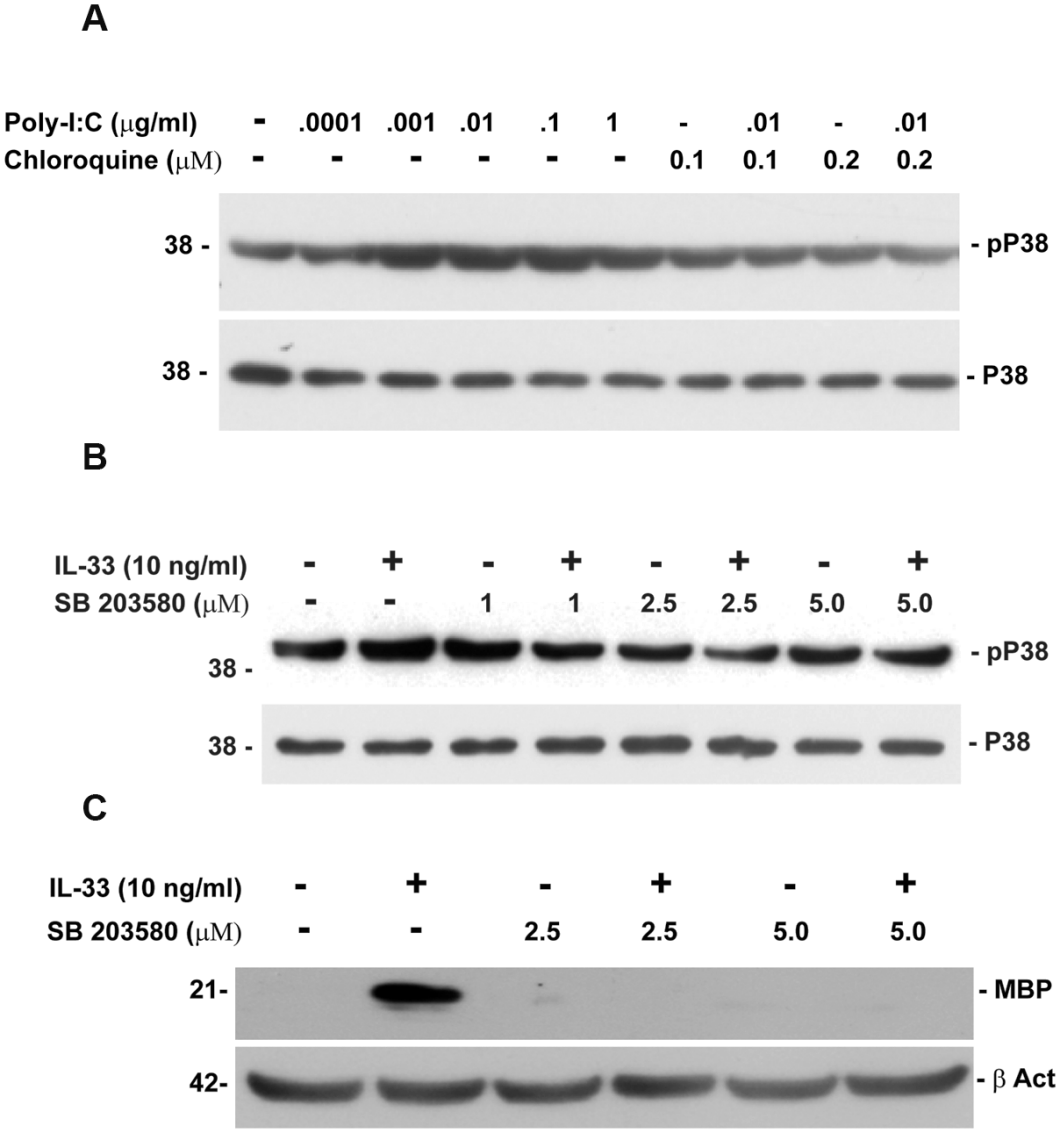
Phosphorylation of p38MAPK in OPC cultured with poly-IC or IL-33 (representative of two experiments). (A) Western blots shows the dose dependent increase in phospho-p38MAPK expression in the presence of Poly-IC and its inhibition by chloroquine; (B) Western blot shows dose dependent increase in P-p38MAPK expression after addition of IL-33 and its inhibition by p38MAPK inhibitor SB203580; (C) inhibition of MBP in OPC cultured with IL-33 in the presence of SB203580.

Previous studies have shown the importance of activation of p38MAPK in inducing myelin basic protein expression in oligodendrocytes [24]. Since, p38MAPK is a signaling molecule which is down stream of TLR3 and the IL-33 receptor, we examined the phosphorylation p38MAPK in OPC following culture with either poly-IC or IL-33 [25]. A dose dependent phosphorylation of p38MAPK was seen in OPC cultured with poly-IC. Phosphorylation was inhibited by chloroquine, a known inhibitor of TLR3 (Fig 6A). To test whether IL-33 mediated increase of MBP in OPC is also regulated by p38MAPK, we examined the expression of MBP in the presence of p38MAPK inhibitor SB 203580. *In vitro* pretreatment of OPC with IL-33 increased the level of MBP by 49.7 ± 17 fold when compared with vehicle treated controls and IL-33 stimulation. Addition of SB 203580 reduced the level of MBP to background levels (Fig 6B and 6C).

### *In vivo* treatment with poly-IC improves remyelination of the CC following injection with lysolecithin

**Fig 7:**
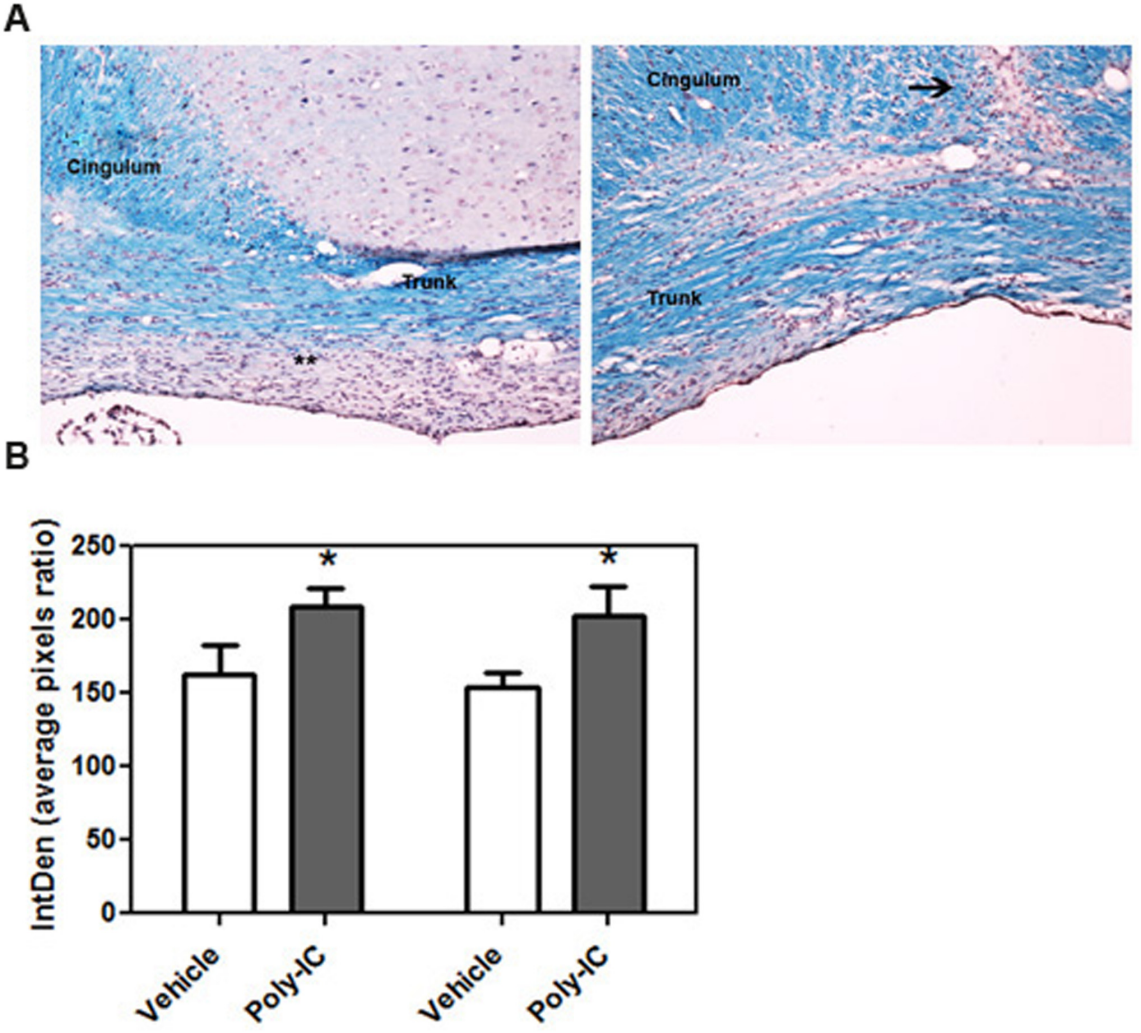
(A) Representative images of corpus callosum of animals injected with lysolecithin and treated with vehicle (left) and poly-IC (right). Paraffin embedded coronal tissue sections were stained with LFB to quantitate myelin at day 21 after vehicle and poly-IC treatment. Animals were injected IC with lysolecithin on day 0 and treated with poly-IC, or equal volume or vehicle (sc injection of 10 μg/Kg/everyday in 400ul saline solution) for 21 days. (B) Bar graphs represent quantitative analysis of myelin staining at day 14 and 21 following lysolecithin injection. Quantitation done from three separate experiments; vehicle = 4 rats for day 14 and 7 rats for day 21 and poly-IC = 4 rats for day 14 and 7 for day 21. *, p<0.05., **, areas of demyelination persists until day 21 post lysolecithin injection. Arrow shows the lysolecithin injection site.

In the gliotoxic model of demyelination induced by lysolecithin, maximal demyelination is seen at about 3 weeks post injection and remyelination is complete within 4–5 weeks. We therefore examined the degree of remyelination in rats injected with lysolecithin and treated daily with poly-IC. We quantitated the amount of myelin present at day 14 and day 21 post injection with lysolecithin. Quantitative analysis of the amount of myelin staining was done with Luxol Fast Blue (LFB). Image analysis of corpus callosum showed that there was 32.8% increased LFB staining in the cingulum and trunk of corpus callosum on day 14 and a 28.7% increase on day 21 in animals which were treated with poly-IC when compared to vehicle treated animals (P<0.02, Fig 7A and 7B).

**Fig 8:**
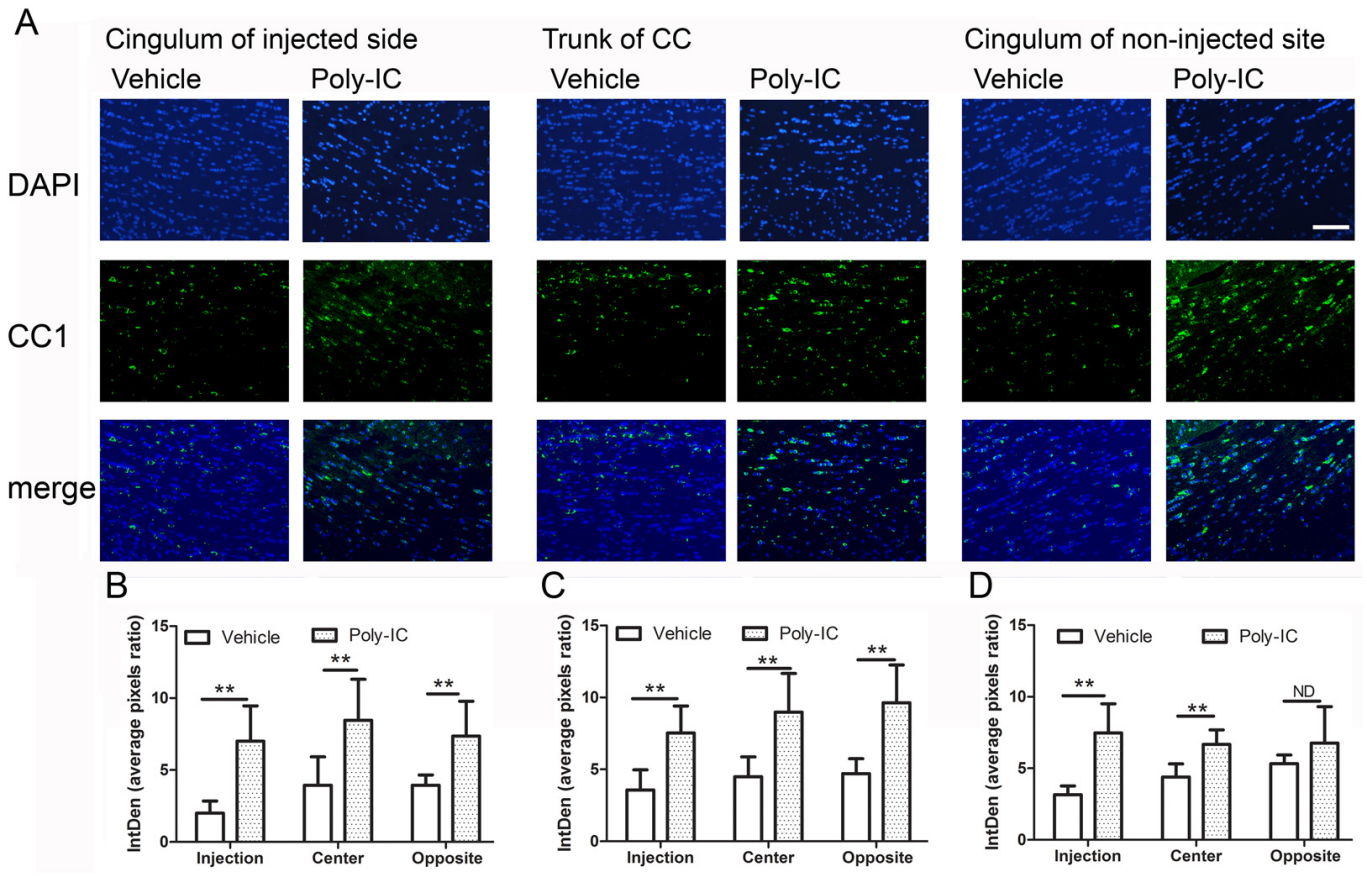
A-D. Poly-IC treatment improves OPC maturation and enhances remyelination in Lysolecithin induces de-myelination rat model. (A) Immunohistochemical staining of CC1 displays a increasing CC1 positive cell number in injection site of Poly-IC treated 14 days post injection. (B, C, D) Quantitative analysis of CC1 expression in the region of cingulum on the side of injection, the trunk of the corpus callosum and on the opposite non injected hemisphere. The analysis were done from 6 animals from poly-IC treated or vehicle treated animals at day 7,14 and 21 (**P<0.01 between vehicle treated and poly-IC treated groups for all regions on days 7,14 and 21).

To further examine the expression of mature myelinating cells in regions which had undergone demyelination, we stained the regions of the corpus callosum for the expression of CC-1 (maturation marker of OPC) and myelin basic protein. The expression of CC-1 was quantitated at the level of the injection site, the trunk of the corpus callosum and the cingulum of the opposite hemisphere. At all three time points and the three different anatomical locations, CC-1 expression was higher when compared to vehicle treated controls (Fig 8A–8D). The quantitated expression levels of CC-1 are as follows: (a) day 7 in poly-IC treated animals, expression in the three anatomical regions (injection site, trunk of corpus callosum and cingulum of the opposite hemisphere) were 7.01±2.45, 8.98±2.69 and 6.67±1.0; in the vehicle treated group the expression levels were 2.01±0.84, 4.49±1.38 and 3.94±0.72; (b) on day 14, poly IC treated animals expression in the three regions of the corpus callosum was 7.73±1.87, 8.98±2.69 and 9.63±2.62. In vehicle treated animals it was 3.57±1.4, 4.49±1.38 and 4.71±1.63; (c) on day 21, expression of poly-IC 7.47±2.04, 6.67±1.0 and 6.76±2.55. In vehicle treated animals CC-1 expression levels were 3.16+0.6, 4.39+0.2 and 5.32+0.61.

**Fig 9:**
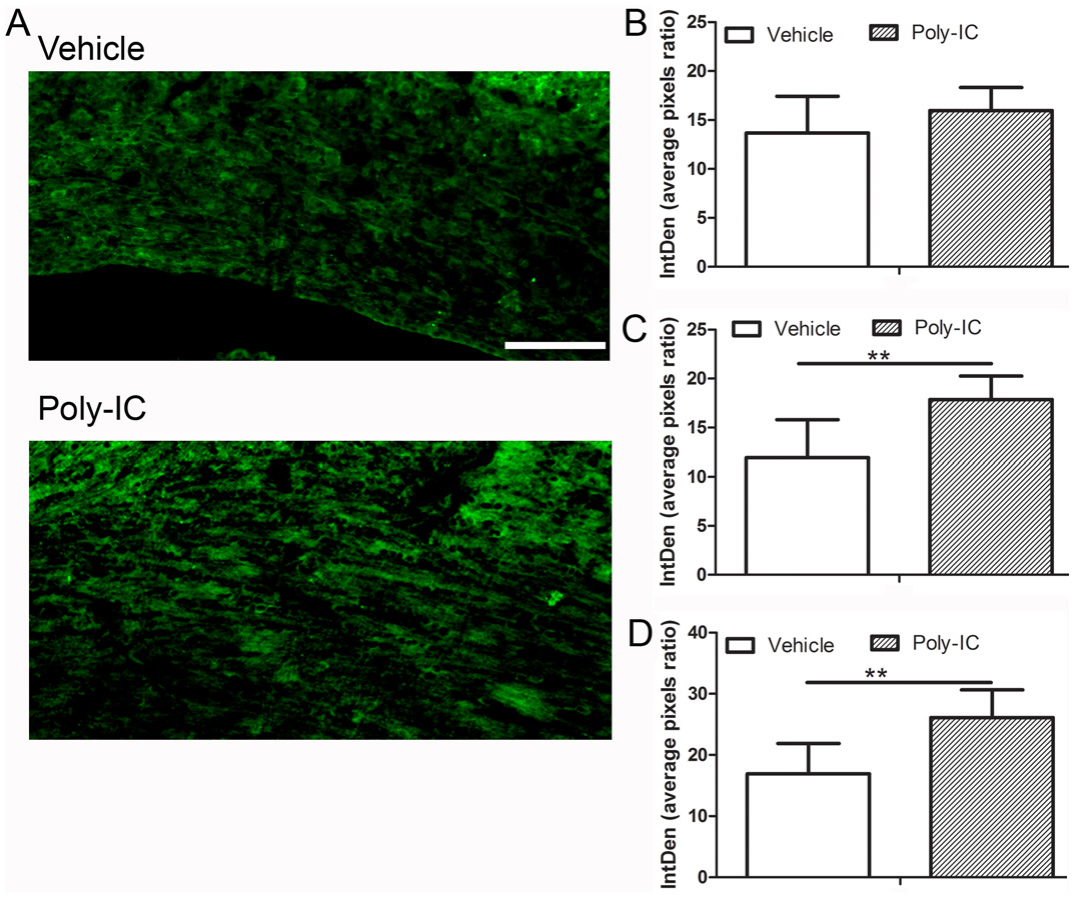
A-D. Immunostaining of MBP in sections in animals whch received poly-IC of animals sacrificed on day 21. Staining of myelin with anti MBP antibodies in a representative section of brain of poly-IC treated and vehicle treated animal (A);, quantitative analysis of MBP expression in corpus callosum on days, 7,14 and 21 post injection with lysolecithin (B-D, n = 6/group, **p<0.01).

We also stained for the expression of myelin basic protein in the regions of the corpus callosum close to the injection site. Increase in MBP expression was seen in poly-IC treated animals when compared to controls on days 14 and 21 but not day 7 (Fig 9). At 14 days the MBP expression was 17.87+2.39 in the poly-IC treated group and 11.92+3.89 in the vehicle group. At day 21, the expression was higher as was 26.1± 4.5 in the treated group an d16.9± 4.6 in the vehicle control (P<0.01).

### *In vivo* treatment with poly-IC increases recruitment of Olig2 expressing OPC in the area of lysolecithin induced demyelination

**Fig 10:**
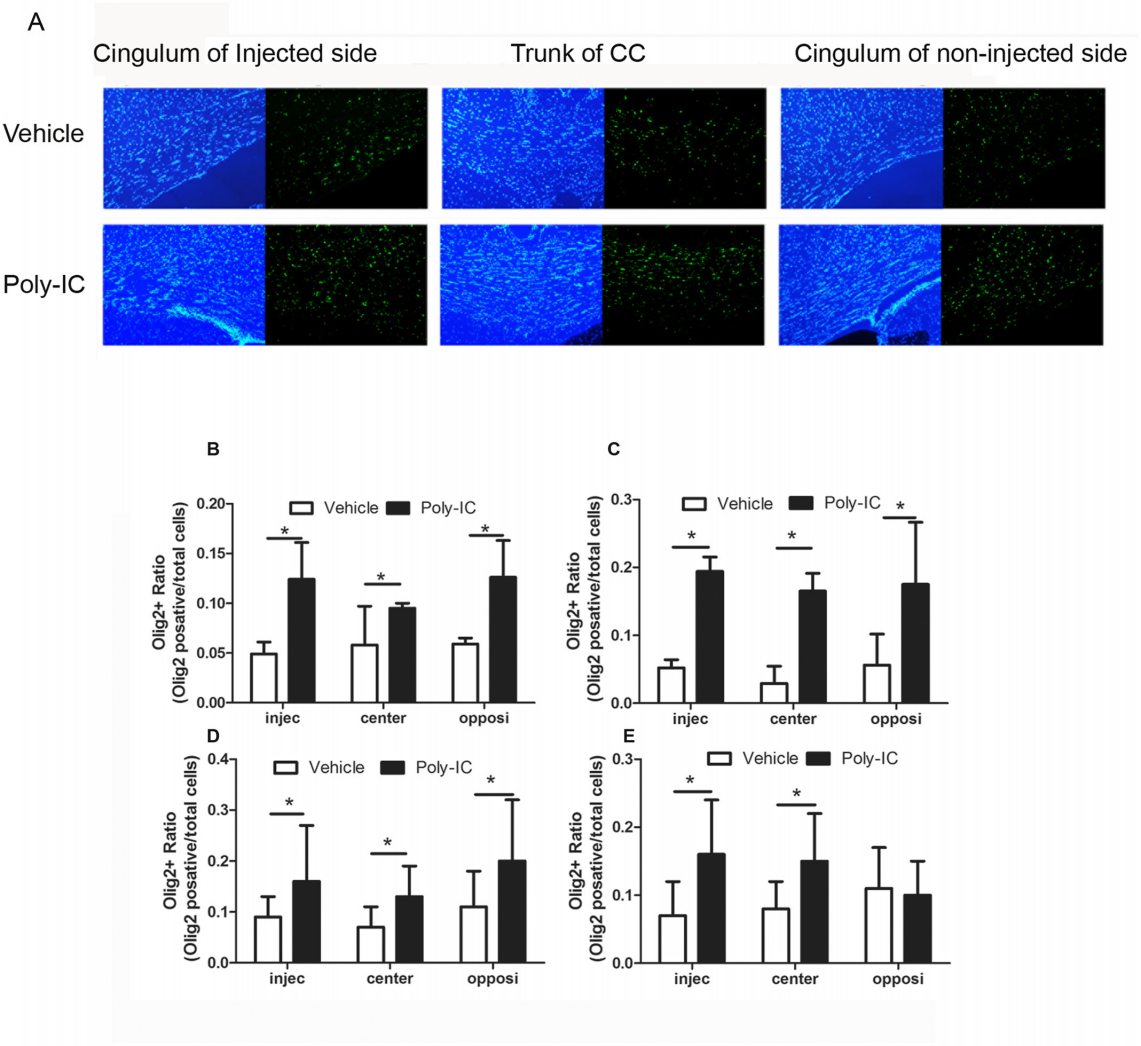
(A-E). Poly-IC treatment recruits Olig2^+^ positive OPCs into the area of corpus callosum (CC) in lysolecithin injected rats; animals were injected on day 0 with lysolecithin and treated daily for 21 days with poly-IC or vehicle (sc injection of 10 μg/Kg/everyday in 400ul saline solution). (A), Immunohistochemical analysis of lysolecithin injected rats treated with poly-IC for 7 d displays more Olig2^+^ OPC in CC. (B, C, D& E) Quantitative analysis of Olig2 expression in CC of brain sections collected on day 3, 7, 14 and 21 post poly-IC treatment respectively. Olig2+ quantification was performed in the cingulum of lysolecithin injection side, trunk of CC and opposite side of the cingulum. Quantitation was done from three separate experiments; vehicle treatment = 3 rats used for day 3, 6 rats for day 7 and 14 and 7 rats for day 21. Poly-IC treatment = 3 rats for day 3, 6 rats for day 7 and 14 and 9 rats for day 21 *, p<0.05.

Since the amount of LFB staining for myelin was seen earlier in animals injected with lysolecithin and treated with poly-IC, we examined if there was a greater recruitment of OPC in regions undergoing demyelination. Coronal sections of the brain were obtained on day 3,7,14 and 21 in animals injected with lysolecithin and treated daily with poly-IC and stained for the presence of Olig2. As shown in Fig 10A, there was increased expression of Olig2 + cells in the corpus callosum, which included the region of the cingulum of the injection site, the trunk and the cingulum of the opposite hemisphere in poly-IC treated animals when compared with vehicle control. Increased expression of Olig2+ cells was seen as early as day 3 post injection and continued until day 21 post injection (one way ANOVA, p, 0.02 on days 3, 7 and 14 and in all three regions between poly-IC treated and vehicle treated animals). Increase in Olig2 expression was not seen white matter regions not in the vicinity of lysolecithin injection. On day 21, statistical difference in Olig2 expression between vehicle and poly IC treated animals was seen only at the site of injection and the trunk, but not in the cingulum of the non-injected contralateral hemisphere (Fig 10B–10E).

### *In vivo* treatment with poly-IC induces the recruitment of IL-33 expressing glial cells in the corpus callosum

**Fig 11:**
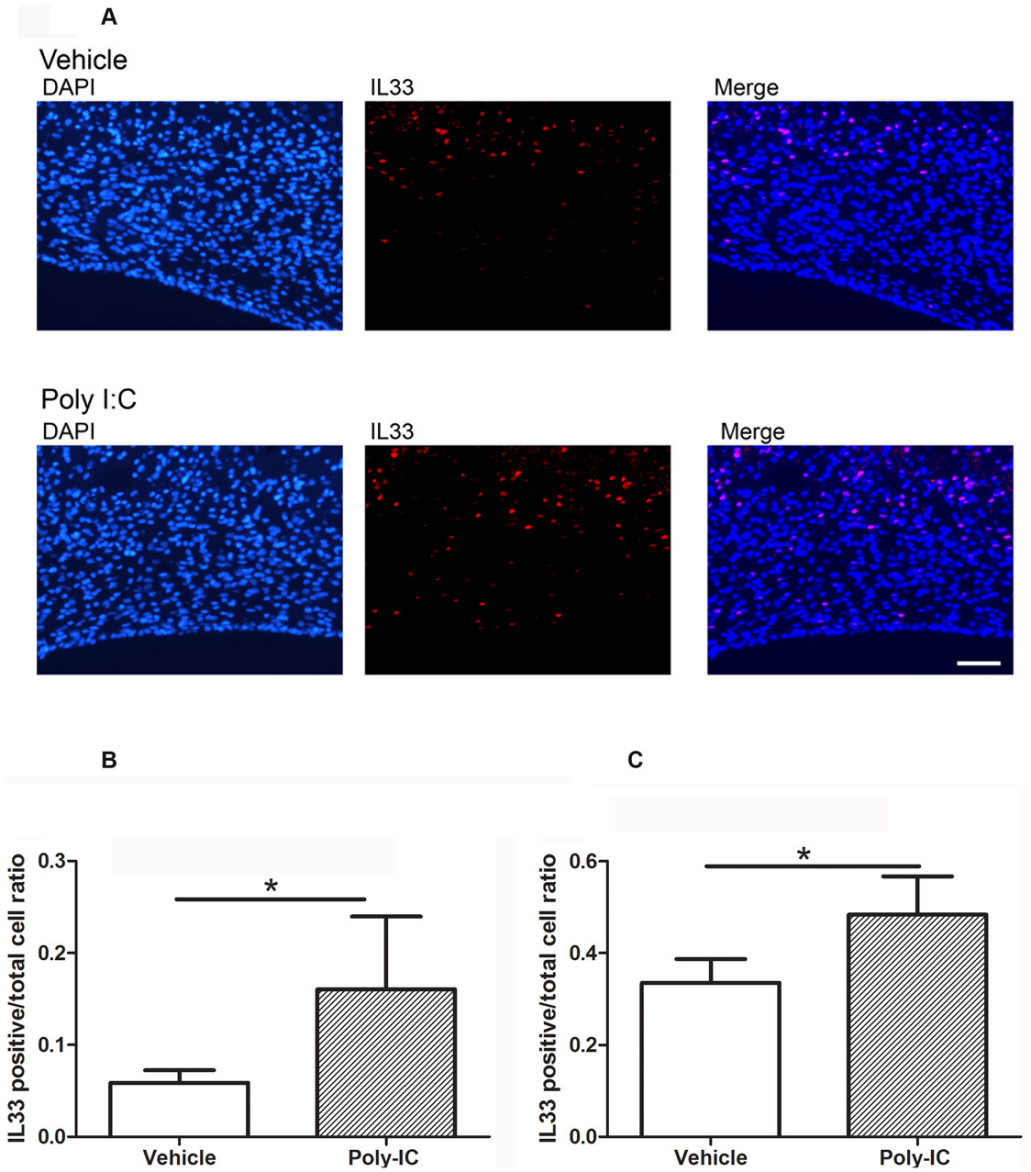
A-C. Poly-IC treatment upregulates IL-33 expression in CC after injection of lysolecithin and treated with poly-IC or vehicle. (A), Immunohistochemical images of rat brain showing the expression of IL-33 in CC. Rats injected with lysolecithin were treated with vehicle or poly-IC for 3 or 7 days respectively. Quantitative analysis of IL-33 performed on day 3 (B) and 7 (C) after lysolecithin injection in animals which received either poly-IC or vehicle.

Since poly-IC induces expression of IL-33 *in vitro*, we examined if *in vivo* treatment with poly-IC will similarly induce IL-33 in regions of demyelination following gliotoxic injury. On day 3 post injection with lysolecithin and treatment with poly-IC, 0.05±0.013% cells were IL-33+, in the vehicle treated group and 0.16 ±0.08% in the poly IC treated group (Fig 11B; p<0.04). On day 7 post lysolecithin injection, expression of IL-33 increased from 0.16 ±0.08% (seen on day 3) to 0.48±0.08% in the poly-IC treated group as compared to an increase from 0.05±0.013% (seen on day 3) to 0.33±0.05% in the controls. (Fig 11B and 11C, p<0.02). By day 14, no IL-33 expressing cells were seen.

**Fig 12:**
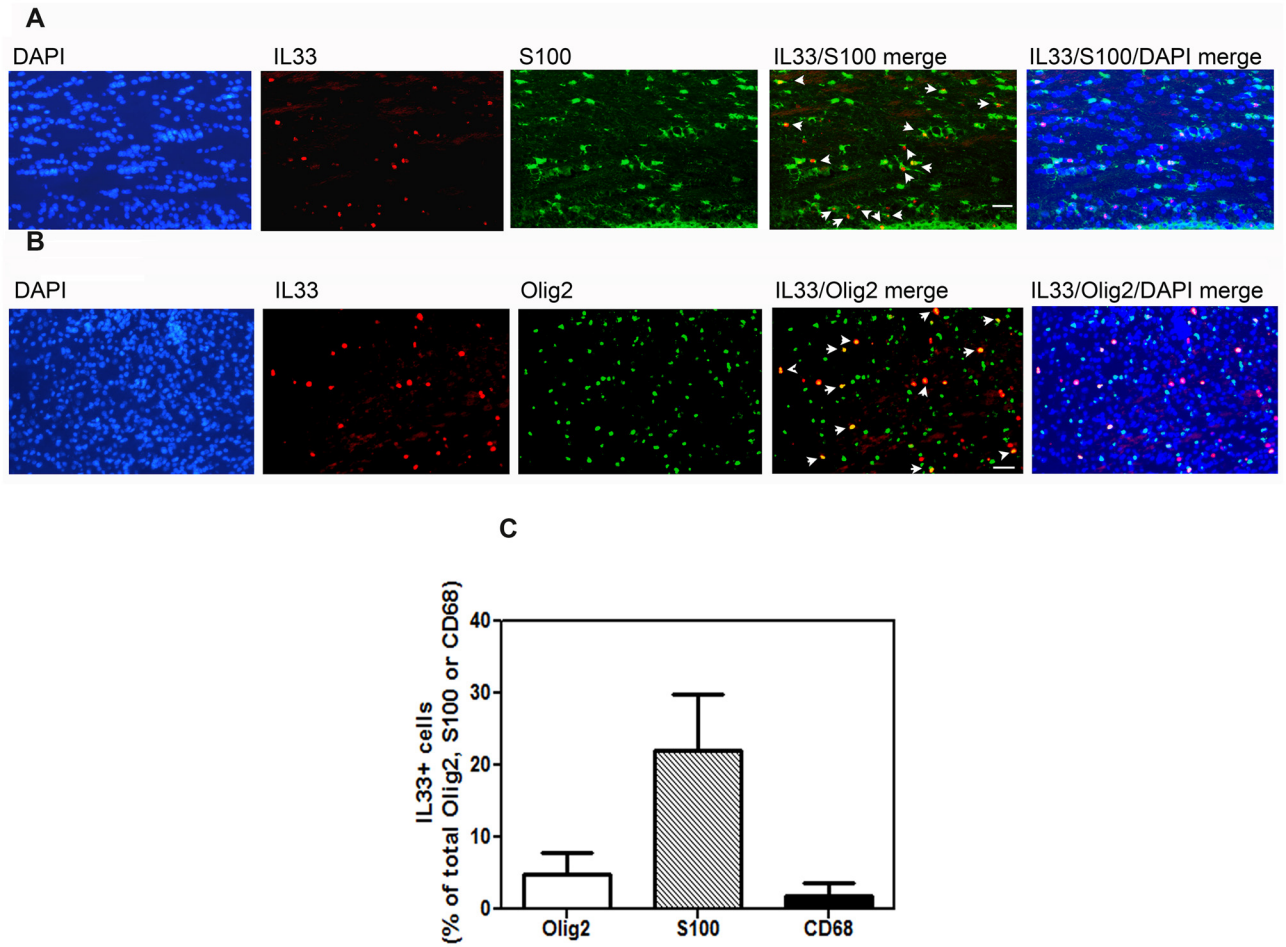
A-C. IL-33 predominantly co-localizes with astrocytes and oligodendrocytes following poly-IC treatment *in vivo*. Immunohistochemical analysis of IL-33 co-localization (white arrows) with the astrocyte marker S100 (A), the oligodendrocyte marker Olig2 (white arrows) (B). (C), Bar graph represents quantitative analyses of percent of cells expressing IL-33 in astrocytes (S100) oligodendrocytes (Olig2+) and CD68 (macrophages).

We also determined the phenotype of cells expressing IL-33 (Fig 12A). The majority of cells which stained with IL-33 were S-100 expressing astrocytes (22±7.7%) (Fig 10A). In contrast, only 4.8 ± 3% of Olig 2 expressing cells (Fig 10B) co-localized with IL-33. IL-33 expression was infrequent in CD-68 staining microglia/macrophages (1.9+1.7%) (Fig 12C).

### IL-33 and poly- IC polarize the macrophages to the M2 phenotype

**Fig 13:**
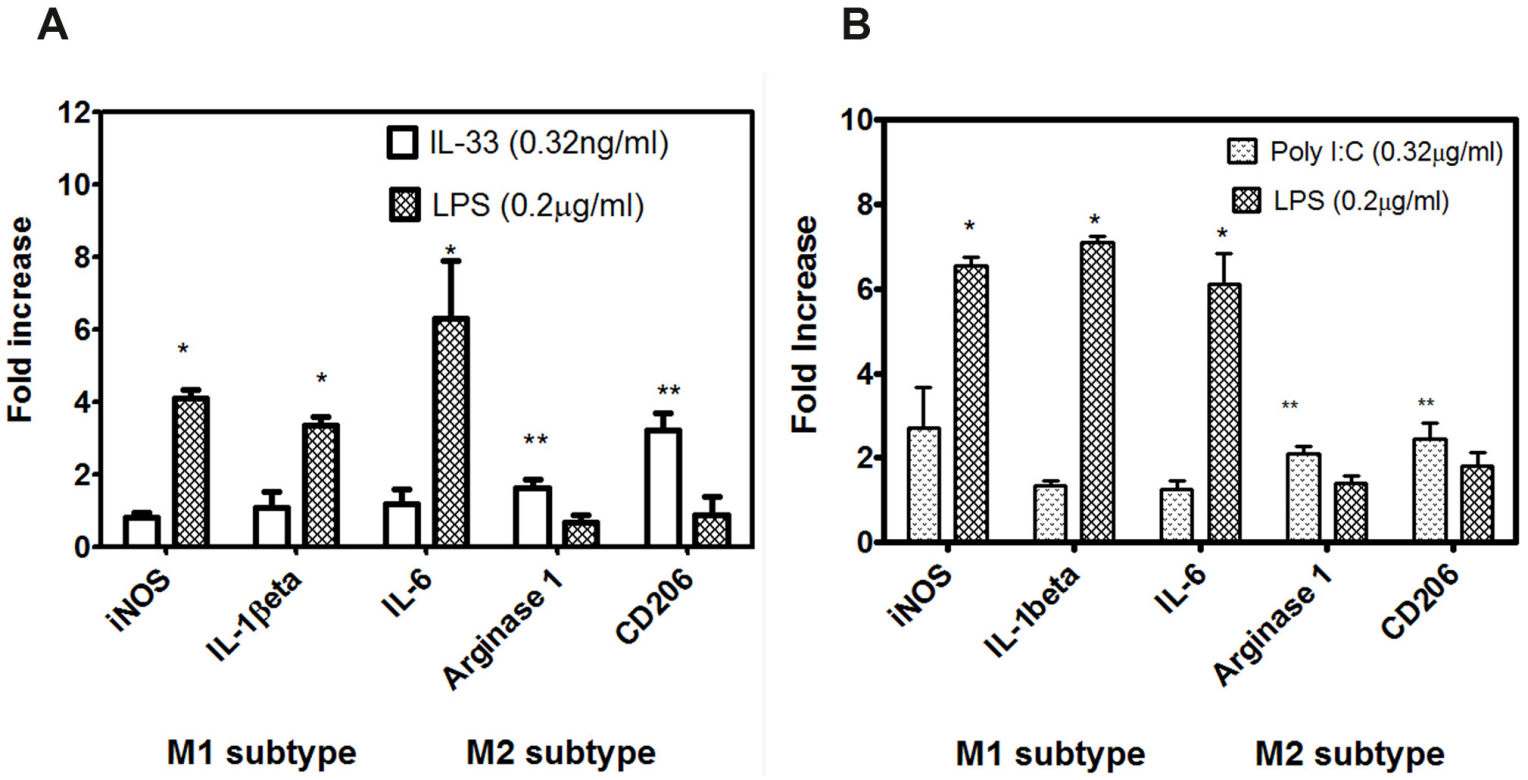
Quantitative RT-PCR analysis of genes which are present in M1 and M2 macrophages after stimulation with IL-33 or LPS left panel, Poly-IC and LPS (right panel); [one way ANOVA, P<0.01 for all M2 genes in IL-33 cultured cells when compared to LPS]; results are expressed as fold increase over unstimulated cells, after normalizing the values to GAPDH.

To demonstrate whether IL-33 influences the polarization of macrophages, we stimulated splenic macrophages in the presence of IL-33 or Poly-IC for 4h and examined for the expression of biomarkers which reflect macrophage activation and polarization. LPS (0.2μg/ml) was used as an inducer of M1 subtype of macrophages. As shown in Fig 11, addition of 0.32 ng/ml of IL-33 to macrophage cultures resulted in an increased expression of M2 markers, such as Arg1 (fold increase 2.11±0.16) and CD206 (2.9+0.38) and were higher when compared to cells stimulated with LPS. Expression of iNOS, IL-1 beta and IL-6 were higher in LPS treated cells compared with cells treated with IL-33. Similarly, addition of poly-IC (0.32ng/ml) to macrophage cultures also resulted in an increase expression of Arg1 and CD206 (2.9 ±0.24 and 3.26±0.5 fold respectively) when compared to cells cultured with LPS. In contrast, splenic macrophages cultured with LPS (0.2μg/ml) showed 5.37±1.6 fold increase of iNOS, 6.6±0.48 fold increase of IL-1β and 9.7±4.5 increase of IL-6. M2 markers (Arg 1 and CD206) expressions were lower following stimulation with LPS, when compared to poly-IC treated cells. LPS (1.0±0.5, 0.59 ± 0.38 respectively) suggesting that LPS treatment skewed the macrophages towards the M1 phenotype, whereas both poly-IC and IL-33 skewed macrophages towards the neuroprotective M2 phenotype (Fig 13).

We also examined the effect on poly-IC treatment on the expression of markers for M1 and M2 macrophages *in vivo*. Previous studies and our supplementary data have shown that IL-33 polarizes macrophages to the M2 phenotype and our experiments on the effect of poly-IC and IL-33 on the polarization of the macrophages.

**Fig 14:**
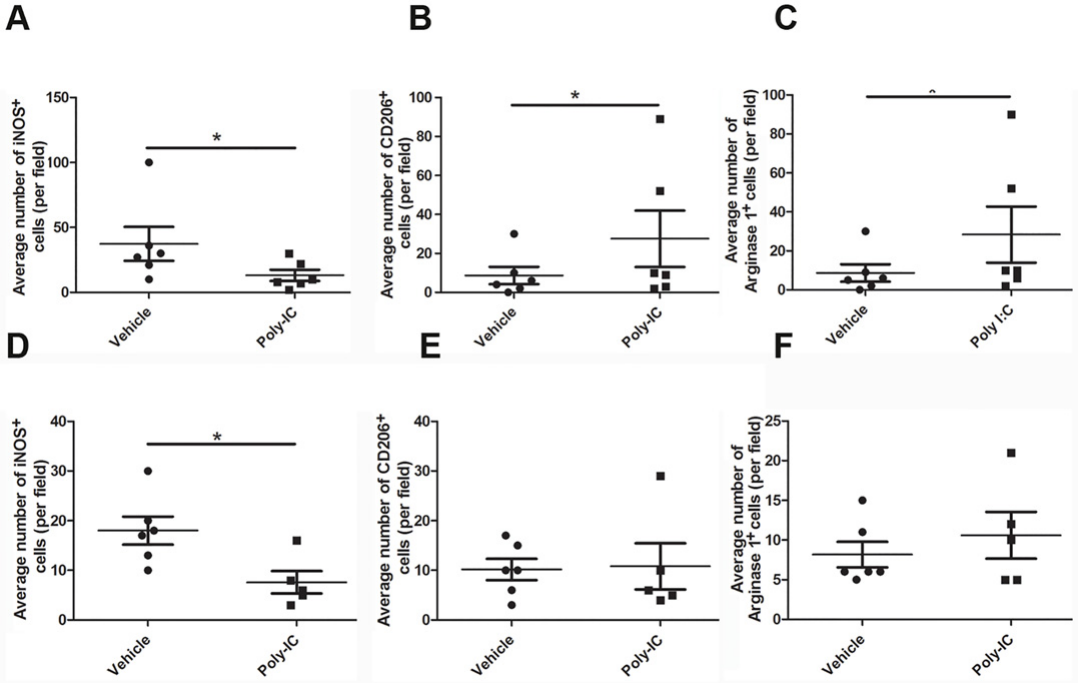
Quantitation of ED1 macrophages expressing iNOS, CD206 or Arginase 1 in the regions of the injection site and trunk of corpus callosum in Poly-IC treated and vehicle treated controls. Results are the number of cells expressing either M1 or M2 marker in the region of the injection site and the adjacent trunk of corpus callosum. A-C, day 14 and D-F, day 21 following injection with lysolecithin; each dot represents one section from poly-IC or vehicle treatment.

We quantitated the expression of iNOS (M1 marker) along with CD206 (M2 marker) and Arginase 1 (M2 marker) in regions of demyelination in animals treated with poly-IC or vehicle following injection with lysolecithin. Quantitative immunohistochemical studies done on day 14 after injection with lysolecithin showed higher expression of Arginase 1 (28.3 ± 14.4) and CD206 (27.5 ± 14.5) in animals treated with poly-IC when compared with vehicle treated controls (8.67 ± 4.5 and 8.7 ± 4.4 respectively) (p<0.05). In contrast, iNOS positive macrophages were found higher in vehicle treatment (37.3 ± 13.0) when compared to poly-IC treated animals (13.2 ± 4.3). On day 21, iNOS expression was higher in vehicle treated controls rats (18.28 ± 2.8) when compared with poly-IC treatment (7.6 ± 2.2). However, there was no difference in the expression of Arginase 1 (8.17 ± 1.62) and CD206 (10.8 ± 4.7) between poly-IC treated animals and vehicle treated controls (Arginase 1, 8.17 ± 1.62; CD206, 10.17 ± 2.15) (Fig 14).

## Discussion

Our study shows that poly-IC and IL-33 (which is induced by poly-IC) enhance the expression of myelin genes and induce differentiation of OPC to mature oligodendrocytes. IL-33 expression in OPC led to a positive feedback autocrine regulation, and increased expression of IL-33. Also, poly-IC administered *in vivo* resulted in increased staining for myelin in lysolecithin induced model of demyelination. The demyelinated regions of the corpus callosum in animals treated with Poly-IC had higher levels of expression of CC-1 and myelin basic protein. The increased staining for myelin was associated with recruitment of Olig2+ oligodendrocytes, increased expression of IL-33 in astrocytes and to a lesser degree in OPC suggesting an ongoing remyelinating process. IL-33 was also seen in animals which did not receive poly-IC, suggesting that the natural process of remyelination which occurred following lysolecithin induced gliotoxic injury could be influenced by IL-33. In contrast to a recent study, we did not see constitutive expression of IL-33 in glial cells in normal animals [17]. The numbers of arginase 1 and CD 206 expressing macrophages were greater in regions undergoing demyelination suggesting that *in vivo* treatment with poly-IC promotes polarization to the M2 subtype of macrophages in animals treated with Poly-IC. The early appearance of IL-33 followed temporally by the appearance of the M2 macrophages suggests that IL-33 is a candidate cytokine involved in polarization of macrophages to the M2 phenotype.

We show that IL-33 acts directly on OPC and promotes expression of myelin genes and therefore likely to influence remyelination. One mechanism by which both poly-IC and IL-33 can induce the activation of myelin genes is through the activation of the p38MAPK pathway. Mitogen activated protein kinases (MAPKs) comprise a family of Ser/Thr specific kinases activated by a diverse array of intra and extracellular signals. P38MAPK is primarily activated by cellular stress and both TLR3 and IL-33 receptors utilize p38 as downstream signaling molecule. In OPC, p38MAPK regulates the myelinating process in the developing brain and is a key step in the induction of lineage dependent progression of oligodendrocytes from immature to mature forms. The mechanism by which p38MAPK, mediates its effect on myelin gene expression is complex and involves the direct regulation of Sox 10 and through the negative regulation of ERK and JNK pathways [24, 26].

Our results are at variance from one published study which suggested that poly-IC was toxic to oligodendrocytes [27]. This report showed that the viability of OPC was affected when poly-IC was added at dose of 1μg/ml or higher and maintained in culture for extended periods of time. We used poly-IC at a concentration of 0.01μg/ml, (which was 100 fold less than those reported) and observed no decrease is cell viability. Induction of MBP was seen following addition of poly-IC at doses as low as 0.01μg/ml. Also, maturation of OPC and polarization of macrophages to the M2 subtype was seen at 0.32μg/ml, suggesting that the toxic effect of poly-IC on OPC and macrophages was most likely dose related.

The mechanism by which IL-33 promotes remyelination was thought to be due to its modulation of macrophage function [17, 28]. Macrophages adopt two broadly exclusive phenotypes, depending upon the stimuli they receive [14, 29, 30]. M1 macrophages are typically pro-inflammatory and contribute to tissue injury by production of cytokines and reactive oxygen species, which impair glial function. M2 macrophages on the other hand, are involved in clearing myelin debris and provide growth factors for recovery [15]. IL-33 polarizes macrophages to the M2 subtype and the therapeutic benefit of IL-33 in EAE and following spinal cord injury has been attributed to the pro-reparative milieu, induced by M2 macrophages. One study identified, Activin-A made by M2 macrophages as a new growth factor involved in remyelination [16]. The activation of myelin genes by poly-IC and IL-33 suggests the presence of a direct effect of TLR and IL-33 signaling on myelination.

**Fig 15:**
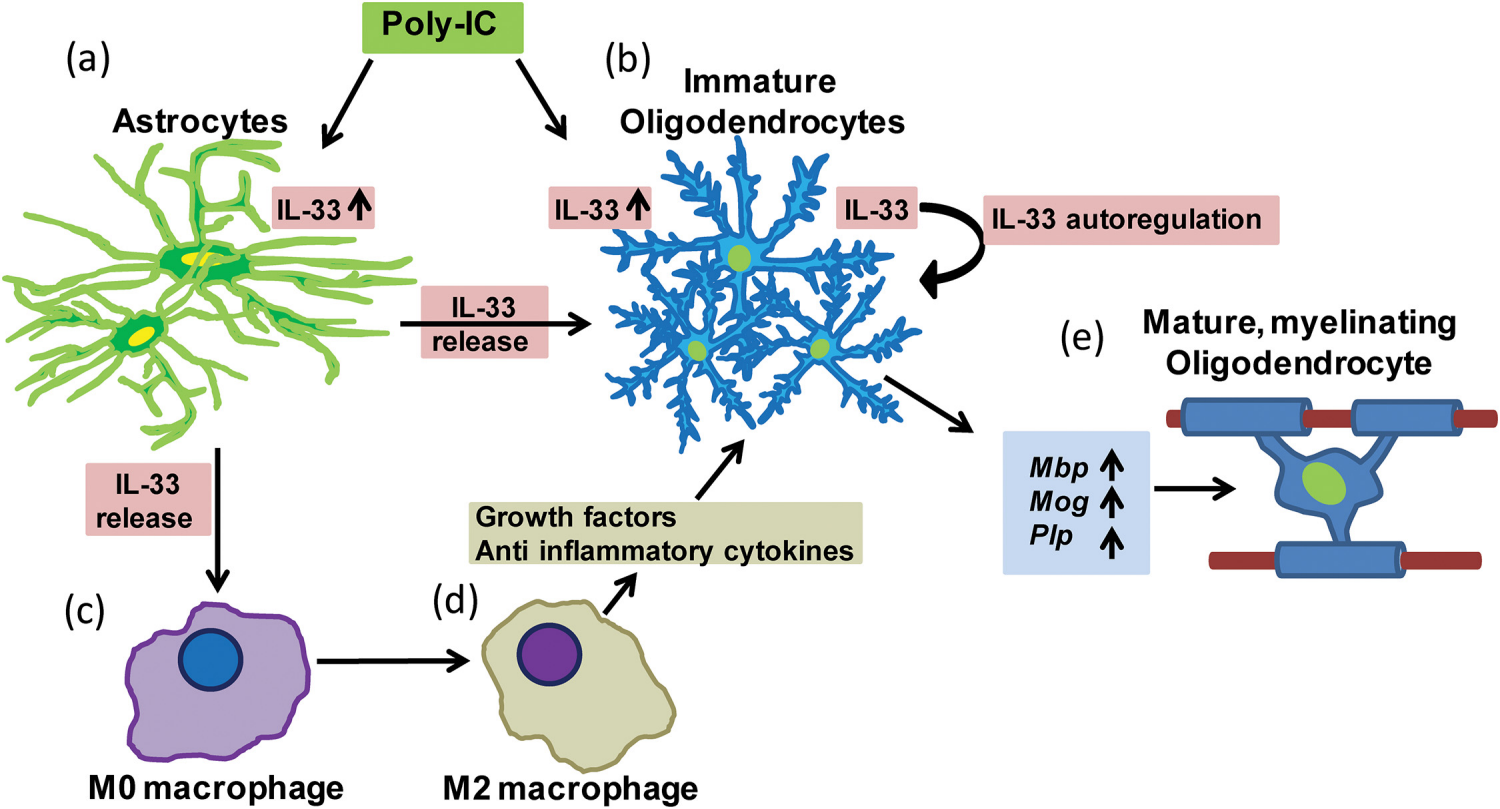
Poly-IC induces IL-33 upregulation and promotes myelin synthesis. Poly-IC induces intranuclear accumulation of IL-33 in astrocytes (a) and in oligodendrocytes (b) leads to expression of myelin genes (e); IL-33 released by OPC amplifies OPC maturation in an autocrine manner (b); IL-33 released from astrocytes and oligodendrocytes promote polarization of macrophages to M2 phenotype (c, d) and promote oligodendrocyte maturation and myelination (e).

Our study suggests that systemic injection of poly-IC has direct effects within the CNS in modulating a neuro-reparative response. Systemic injection of poly-IC has also shown to reduce ischemia/reperfusion injury when administered after the ischemic event and hence a direct effect of poly-IC on glial cells is very likely [31]. We propose that poly-IC and IL-33 by acting of oligodendrocytes, can directly promote *activation of myelin genes required for remyelination*. The remyelinating functions of poly-IC can be effected by a direct activation of OPC or indirectly through activation of IL-33 induced in OPC or on astrocytes (Fig 15). Although expression or IL-33 in CD65 cells in regions of demyelination was low, we cannot exclude the effect of M2 macrophages induced following subcutaneous injection with poly-IC in promoting remyelination.

Poly-IC is a synthetic mimic of viral double stranded RNA which interacts with TLR3 and cytosolic RNA helicase MDA-5 [7, 9]. We have reported increased expression of IL-33 following *in vitro* culture of peripheral blood mononuclear cells (PBMC) with poly-IC [13]. IL-33 is a 30kDa protein and the most recently discovered member of the IL-1 cytokine gene family [32, 33]. Like IL-1β and IL-18, IL-33 is synthesized with the typical IL-1 family 12-stranded b-trefoil C-terminal cytokine domain. Unlike IL-1 β and IL-18, IL-33 possesses a unique N-terminal nuclear localization sequence and chromatin-binding motif and lacks a secretion signaling sequence [28, 34]. Therefore, IL-33 is mainly located in the nucleus and is released upon necrosis or death of the cell [35]. Although TLR3 is expressed in astrocytes and oligodendrocytes, the induction of IL-33 was seen at higher levels in astrocytes when compared with other glial cells. In this context, the reasons for the rare expression of IL-33 in macrophages while a greater expression in astrocytes and oligodendrocytes in areas of demyelination is not known, since macrophages are known to express TLR3 receptors.

Since IL-33 is exclusively present in the nucleus, the mechanism by which poly-IC induces IL-33 and enhances the expression of myelin is not clear. IL-33 is recognized as an “alarmin”, serving as a host response to pathogens and the release of IL-33 is caused by either cell death or necrosis. One possible mechanism by which IL-33 can be released in the absence of cell death is by activation of purinergic signaling pathways [36]. Although ATP is a well-known source of energy, it also functions as a signaling molecule acting through specific purinergic receptors. In the CNS, astrocytes and oligodendrocytes express the adenosine receptor [37]. Addition of adenosine to *in vitro* culture of astrocytes, stimulated to express IL-33, leads to release of IL-33 and its presence can be measured in culture supernatants [38]. It is therefore conceivable that in an inflammatory milieu, the presence of adenosine could lead to release of IL-33 localized in the astrocytes, into the extracellular space, wherein it could bind and signal through its natural ligand, IL-33 receptor on oligodendrocytes and induce myelin gene expression (ST2/ IL1RAcP) [33, 39, 40]. IL-33 released in the extracellular space could also influence the polarization of macrophages to the M2 phenotype and enhance the recruitment of OPC and promote myelination (Fig 15).

Although both IL-33 and poly-IC, induce the activation of myelin genes, it is not clear if the induction of myelin genes by poly-IC is mediated exclusively by the induction of IL-33. We cannot exclude the existence of other pathways following activation of TLR3 which can positively regulate myelin gene expression and myelin repair. Earlier studies had shown that intracerebral injection of poly-IC has shown to induce beta interferon and promote recovery from EAE [11] [41].

IL-33, can be added to the list of other pro-inflammatory cytokines which have shown to positively regulate myelin gene expression [42]. These include GP130 family of protein, IL-6, IL-11, IL-27, oncostatin M (OSM), leukemia inhibitory factor (LIF), cardiotrophin 1(CT-1), and ciliary neurotrophic factor (CNTF). LIF and CNTF have been shown to protect mature oligodendrocytes from demyelination and also enhance remyelination, and exogenous addition of LIF or CNTF has also been used successfully to limit the consequences of oligodendrocyte damage [43–45].

## Conclusions

Our study suggests that poly-IC, acting either directly or indirectly through the synthesis of IL-33 may be a candidate molecule for use as a therapeutic agent in demyelinating disease. Poly-IC has been used in a limited clinical trial in MS. In a pilot trial of poly-IC in MS, patients tolerated the drug well and there was modest degree of stabilization of the disease [46–48]. Our study suggests that the neuro-reparative features of poly-IC and IL-33 will have clinical applications in the treatment of demyelinating disease.
